# Supplementary material for: Factors Associated with Smoke-Free Pregnancy among Aboriginal and Torres Strait Women and Their Experience of Quitting Smoking in Pregnancy: A Mixed Method Cross-Sectional Study
Source: Int J Environ Res Public Health. 2021 Oct 26;18(21):11240. doi: 10.3390/ijerph182111240 (PMC8583423; doi:10.3390/ijerph182111240)
Supplement: Supplementary file 1 [file ijerph-18-11240-s001.zip › ijerph-1377994-supplementary.pdf]

Table S1: Survey items analysed

Questions	Comments	Variable construction
**Eligibility**		
By selecting "yes", you are agreeing to participate in this survey, and have read and understood the Participant Information Sheet<br>○ Yes<br>○ No	*Sheet provided as PDF link*<br>*No selection causes termination of survey*	-
Please select gender<br>○ Female<br>○ Male<br>○ Other (prefer not to say)	*Male or Other selection causes termination of survey*	-
Are you Aboriginal and/or Torres Strait Islander?<br>○ No<br>○ Yes, Aboriginal<br>○ Yes, Torres Strait Islander<br>○ Yes, Aboriginal and Torres Strait Islander	*No selection causes termination of survey*	-
What is your age?		Age group<br>16-25<br>26-36<br>36 and above
**Demographics**		
1. Would you say you are:<br>○ A current smoker at least one cig per day<br>○ A current smoker but less than one cig per day (including 'social smoker' or occasional smoker)<br>○ An ex-smoker who used to smoked at least one cigarette per day<br>○ An ex-smoker who used to smoked less than one cigarette per day<br>○ Someone who has never smoked, or never smoked regularly	*End survey if someone who has never smoked, or never smoked regularly*	Smoking status<br>Current smokers =<br>○ A current smoker at least one cig per day<br>○ A current smoker but less than one cig per day (including 'social smoker' or occasional smoker)<br>Ex-smokers =<br>○ An ex-smoker who used to smoked at least one cigarette per day

		○ An ex-smoker who used to smoked less than one cigarette per day
2. What state do you live in?<br><br>○ NSW<br>○ QLD<br>○ SA<br>○ NT<br>○ WA<br>○ TAS<br>○ ACT<br>○ VIC		
3. What is your post code?		Geographic remoteness based on Australian Statistical Geography Standard (ASGS)<br>○ Urban<br>○ Regional/Remote
4. Do you use an Aboriginal Health Service(s)?<br><br>○ Yes<br>○ No		AHS use =<br>○ Yes<br>○ No
5. Which Aboriginal Medical Service do you use?	*Shown if answered Yes for question 5*<br>*Shown as an optional response*	
6. What education level have you completed?<br><br>○ Primary school, or up to year 9<br>○ Year 10-11<br>○ Year 12<br>○ Current student at university/TAFE/apprentice<br>○ Trade certificate<br>○ University degree		Education =<br>○ Up to Year 11<br>○ Year 12<br>○ Current student at university/TAFE/apprentice<br>○ Trade certificate<br>○ University degree

7. How many children currently live in your household? (if you are pregnant and have children living in your household, please select both)<br><br>○ None<br>○ 1-2<br>○ 3 or more<br>○ I'm currently pregnant	*Multiselect option*<br>*If selection of "none" participant is unable to select 1-2, or 3 or more.*	Have children in the household =<br>○ None<br>○ 1-2<br>○ 3 or more<br>Pregnant =<br>○ I'm currently pregnant<br>• Yes<br>• No
8. How old is each child? eg: 12, 3, 7	*Only shown if previous question indicated children were living in the house*	
**"These questions are about smoking"**	*Questions only shown if indicated currently smoking (in any frequency) in question 1.*	
9. How many cigarettes do you usually smoke per day (on the days you smoke)?		Heaviness of smoking index (HSI)<br>Q1. Number of cigarettes smoked per day<br>○ 1–10 (0)<br>○ 11–20 (1)<br>○ 21–30 (2)<br>○ 31+ (3)
10. On the mornings that you have a smoke, how soon do you have it after waking up?<br><br>○ More than 60 minutes<br>○ 31-60 minutes<br>○ 6-30 minutes<br>○ Within 5 minutes		Heaviness of smoking index (HSI)<br>Q1. On the mornings that you have a smoke, how soon do you<br>have it after waking up?<br>○ less than/equal to 5 (3)<br>○ 6–30 (2)<br>○ 31–60, (1) and<br>○ 61 + min (0)
11. HSI[1] score and categories		HSI score (Q1+Q2)

[1] The HSI consists of two questions: a) "How many cigarettes do you usually smoke per day (on the days you smoke)?", and b) "On the morning that you have a smoke, how soon do you have it after waking up?" The response options for the latter question are (1) More than 60 minutes; (2) 31-60 minutes; (3) 6-30 minutes; and (4) Within 5 minutes. The HSI score ranges between 0 and 6. This score is then classified into three dependence categories: low dependence (scores between 0 and 2); moderate dependence (scores of 3 and 4) and high dependence (scores of 5 and 6). Data were also collected on frequency of urges to smoke (FUTS) (in the last 24 hours) and strength of urges to smoke (SUTS), both have been previously used with

		○ low dependence (scores between 0 and 2);<br>○ moderate dependence (scores of 3 and 4) and<br>○ high dependence (scores of 5 and 6).
12. How much of the time have you felt the urge to smoke in the last 24 hours?<br><br>○ Not at all<br>○ A little of the time<br>○ Some of the time<br>○ A lot of the time<br>○ Almost all the time<br>○ All the time		Frequency of urges to smoke (FUTS)<br>Low<br>○ Not at all (0)<br>○ A little of the time (1)<br>○ Some of the time (2)<br>High<br>○ A lot of the time (3)<br>○ Almost all the time (4)<br>○ All the time (5)
13. In general, how strong are your urges to smoke (in the last 24 hrs)?<br><br>○ No urges<br>○ Slight<br>○ Moderate<br>○ Strong<br>○ Very strong<br>○ Extremely strong		Strength of urges to smoke (SUTS)<br>Low<br>○ No urges (0)<br>○ Slight (1)<br>○ Moderate (2)<br>High<br>○ Strong (3)<br>○ Very strong (4)<br>○ Extremely strong (5)
**"Now some questions about your experiences with quitting"**		
14. In the last month, have you tried to cut down the number of smokes you have each day?	*Shown if indicated in Q1 to be currently smoking in any frequency.*	Cut down in the last month (for current smokers only)<br>○ Yes

Aboriginal communities. FUTS was measured by a single question: "How much of the time have you felt the urge to smoke in the last 24 h?". The response options are Not at all (0); A little of the time; (1) Some of the time; (2) A lot of the time (3); Almost all the time (4); and All the time (5). Participants were grouped into two categories; those scoring between 0-2 were categorized as low FUTS and those who scored between 3-5 were considered high FUTS. Likewise, SUTS was measured by a single question: "In general, how strong are your urges to smoke (in the last 24 h)?". Scores were categories as low (0-2) and high (3-5).

○ Yes<br>○ No		○ No
15. How long ago was your last quitting attempt?<br><br>○ Days<br>○ Weeks<br>○ Months<br>○ Never		How long ago was the latest quit attempt<br>○ Days<br>○ Weeks<br>○ Months
16. Were any of your quit attempts because you were pregnant?<br><br>○ Yes<br>○ No	*Shown if Q15 indicated previous attempt at quitting*	Pregnancy-related quit attempt<br>○ Yes<br>○ No
17. Were you able to stay smoke-free the whole way through your pregnancy?<br><br>○ Stayed smoke-free whole way through<br>○ Smoked occasionally<br>○ Smoke-free for several months<br>○ Cut down<br>○ Other (please tell me more_______)	*Shown if Q16 indicated previous attempt at quitting a result of pregnancy.*	Smoke-free pregnancy<br>○ Yes<br>• Stayed smoke-free whole way through<br><br>○ No<br>• Smoked occasionally<br>• Smoke-free for several months<br>• Cut down<br>• Other (please tell me more_______)
18. Can you tell me a bit about trying to quit when pregnant?	*Shown if Q16 indicated previous attempt at quitting a result of pregnancy.*	Qualitative data
19. Of all the times you tried to quit smoking, what was the longest period you stayed completely off the smokes for?<br><br>○ Hours<br>○ Days<br>○ Weeks	*Shown if Q15 indicated previous quitting attempt*	Longest quit attempt ever<br>○ Hours<br>○ Days<br>○ Weeks<br>○ Months<br>○ Years<br>○ Don't know

○ Months<br>○ Years<br>○ Don't know		
20. On your most recent quit attempt, did you stop smoking suddenly or did you gradually cut down your smokes?<br><br>○ Stopped suddenly<br>○ Cut down gradually	*Shown if Q15 indicated previous quitting attempt*	Nature of last quit attempt<br>○ Stopped suddenly<br>○ Cut down gradually
21. Have you ever used any type of stop-smoking medications?<br><br>○ Yes<br>○ No	*Shown if Q14 indicated reduction attempt*	Ever tried medication for quitting<br>○ Yes<br>○ No

Table S2: Description of participants' experience of smoke-free pregnancies

Sl	Experience of quitting smoking during pregnancy	Stayed smoke-free	Did not stay smoke-free			
		Stayed smoke-free the whole way through	Smoked occasionally	Smoke-free for several months	Cut down	Other
1	Easier because smoking Made me feel sick		√			
2	I did for the first 6 months but smoked in my last trimester					√
3	I couldn't quit I could only cut down				√	
4	Was stressful				√	
5	My morning sickness was aggravated by the smell of smoke so I didn't smoke and couldn't be around smokers		√			
6	Changed routine. Had pre-natal depression so that made it hard				√	
7	Had maybe one a week			√	√	
8	It was hard but doable		√			
9	I quit as soon as I found out with my first and didn't start again until my 3rd child was 3	√				
10	It was easier because I suffered morning sickness and having a smoke made it worse		√	√	√	
11	It was hard at first but stopped after 3-4 months into pregnancy and only smoked when I was stressing out.		√	√		
12	Health of the unborn babies hard being in a DV relationship		√	√		
13	Due to stress out drove me more to smoke		√			
14	It was easy first time, bit harder the next 3 times		√	√		
15	I didn't. I quit after I gave birth to my twins for 8 moths		√			
16	Ok		√			
17	Used NRT to quit at 4 months.			√		
18	Quitting while pregnant was easy, just wanted to give my child the best start to life.	√				
19	Some days it felt like you need a smoke to relax or clam you down and some days it would make you sick depending on what you felt like when you wake up		√	√		
20	I quit for the first few months, I started stressing at work midway through my pregnancy and started smoking again.		√			
21	With my first I was advised to continue to smoke as trying to quit was putting too much stress on my body and could cause loss of baby, I was told to cut down, which I did. Second pregnancy I quit altogether and lost my twins at 18 weeks, 3rd and 4th I quit in the last few months of pregnancy			√	√	
22	I was a non-smoker for my first 3 pregnancies then started up again when my youngest was 2 so I didn't want to be a pregnant smoker					√
23	I didnt want to make my baby sick or cause any health problems so I stopped smoking			√		

Sl	Experience of quitting smoking during pregnancy	Stayed smoke-free	Did not stay smoke-free			
		Stayed smoke-free the whole way through	Smoked occasionally	Smoke-free for several months	Cut down	Other
24	It was hard and Dr told me not to quit straight out cause it could stress me out and make me lose baby		√			
25	It was stressful due to my living situation		√	√		
26	Very stressful. Abusive relationship made it very difficult due to stress. Cut down to maybe 4 a day each time.				√	
27	Gave up after nausea started. It made me throw up			√		
28	For the babies health				√	
29	i just couldn’t because of the mob I was running with			√		
30	Went cold turkey the day I found out, would have a urge to smoke for a week or two but 1 drag and it would make me vomit.	√				
31	Found out easy	√				
32	Just cut myself right back to 5 or under a day				√	
33	Health risk			√		
34	I can quit but my husband is struggling	√				
35	I quit cold turkey	√				
36	It gave me a lot of drive to quit, but with so many people around me smoking. I didn't have the will power.		√			
37	gave up after a week of finding out I was pregnant	√				
38	It hard				√	
39	I tried to quit on numerous occasions whilst pregnant, but I'd get extremely agitated if I didn't have a smoke.				√	
40	Quit through 1 pregnancy, cut down with subsequent pregnancies				√	
41	I found it easy	√				
42	I think it to was down to a mixture of guilt and morning sickness turning me off cigarettes, I couldn't stand the smell of it. With the 1st child pregnancy I only had morning sickness for a short while and started smoking when it eases off. With the 2nd pregnancy I had morning sickness throughout the whole pregnancy and serious health issues during and after, I remained smoke free for 2 yrs. before taking it back up					√
43	didn’t work			√		
44	It was hard		√			
45	Just gave up	√				
46	Went from having 20+ to only 2 a day		√			
47	Cut down but couldn’t quit				√	
48	It was hard but so much easier for me to quit when it's my child's health at risk	√				
49	As my pregnancy was unexpected I was forced to try quit cold turkey				√	
50	It was easy because I had morning sickness but would occasionally have one very few weeks.		√	√	√	

Sl	Experience of quitting smoking during pregnancy	Stayed smoke-free	Did not stay smoke-free			
		Stayed smoke-free the whole way through	Smoked occasionally	Smoke-free for several months	Cut down	Other
51	It was hard				√	
52	Gave up smoking Tobacco while pregnant but smoked cannabis for extreme Nausea & vomiting		√			
53	Tried patches and gum with last pregnancy, was able to cut down but not stop. Couldn't stop for my other two pregnancies either. Had a lot of stress to deal with relating to the pregnancy. Gave up alcohol while pregnant but was too hard to stop smoking as well.				√	
54	With it being my first pregnancy and having no support, it was quite hard. I smoked to release my stress.		√			
55	It was difficult but I did it so my baby would grow and be healthy		√			
56	Easy made me feel sick		√			
57	Cold turkey			√		
58	Easy.	√				
59	it was harder because your stressed out way more than usual because of hormones. and it was kinda a craving for me			√		
60	I think I body rejected it I just couldn't stand the smell of smoke	√				
61	Hard. Cut down		√			
62	Cos I didn't want my baby to have health issues or affects from my smoking	√				
63	I cut down to half of a cigarette a day, and now I have given up for 3 year's				√	
64	Quit cold turkey	√				
65	I wanted to to be smoke free for my baby & for my own health	√				
66	Just gave it up and never brought another packet since	√				
67	We hard but was very sick	√				
68	Was easy for me because it made me physically sick	√				
69	As soon as I heard the babies heart beat I quit	√				
70	I struggled at times	√		√		
71	I quit pretty easy with the first 2 kids, but unfortunately was unable to quit with my final pregnancy	√		√		
72	Just did it					√
73	It was hard at first, but as my pregnancy progressed I couldn't stand the smell and just quit	√				
74	It was the easiest time ever to quit - my body rejected me smoking - I just stopped and every time I tried to smoke I would gag	√				
75	went cold turkey as soon as I found out	√				
76	Yes		√			
77	It was really hard but I eventually got there by keeping my self occupied on something self other then smoking					√
78	Quit at about 2 months with both pregnancies			√		

Sl	Experience of quitting smoking during pregnancy	Stayed smoke-free	Did not stay smoke-free			
		Stayed smoke-free the whole way through	Smoked occasionally	Smoke-free for several months	Cut down	Other
79	Harder than I expected. Cold turkey	√				
80	I quit when trying to get pregnant	√				
81	It was hard at first so I cut down in the beginning until I finally quit it made it easier to quit knowing I was growing a bub	√				
82	It was pretty simple for me, found out I was pregnant and threw them away. I was so morning sick that I didn't even notice the withdrawals. Only problem was at the time is that I kept taking it back up after the each child was around 3	√				
83	Its very difficult giving up a habit especially smoking while pregnant but just know that your baby isn't getting the enough growth and development as a baby who is not inhaling smoke.		√			
84	I was too stressed and decided it would be better for me to cut down instead.				√	√
85	Was a way to keep my baby safe and also help me kick the habit			√		
86	Its hard but I wanted what was best for my baby		√			
87	Its hard to do whilst pregnant. First and second I smoked most the way through cutting down. Third I gave up for months but not all. Last I gave up before getting pregnant.	√		√	√	√
88	Quit soon I found out I was pregnant	√				
89	Only had one here and there		√			
90	It was difficult, I felt big shame			√	√	
91	As soon as I knew I was pregnant I quit I didn't want to harm my baby	√				
92	It was easy because I was thinking of my baby only 1 pregnancy I had trouble giving up it was because I was under severe stress	√				
93	If was easy very bad morning sickness	√				
94	It was a stressful time for me		√			
95	My Bub came first	√				
96	Quit "cold turkey"	√				
97	Quitting was easy through pregnancy, I listened to my body's need	√				
98	no	√				
99	As soon as I found out I wanted to quit for the health of my baby and myself	√				
100	Was a necessity	√				
101	I stopped immediately	√				
102	no	√				
103	Stopped smoking the day I found out	√				
